# Mr. Hardman, on the Sponge Pessary

**Published:** 1806-03

**Authors:** Edw. Hardman

**Affiliations:** Manchester


### *To the Editors of the Medical and Phyfical Journal*

GENTLEMEN,

IT is with infinite pleasure, I acknowledge my obligations to Mr. G. P. Dawson, of Sunderland, for his valuable Communication respecting the use of the Sponge Pessary, mentioned in the 72d Number of your Journal, for February, 1805; by the use of which I have been enabled to give relief to two females, with procedentia uteri, who have been made comfortable to themselves for several months past, and who could not possibly endure the excruciating pain, occasioned either by the cork or wood pessary; I am in hopes other practioners, actuated by the same

 laudable laudable motives as Mr. D. will be induced to follow an example so truly beneficial to society.

I am, &c.

*Manchester, Feb.* 5, 1806. EDW. HARDMAN.

